# The Effect of Alumina-Rich Spinel Exsolution on the Mechanical Property of Calcium Aluminate Cement-Bonded Corundum Castables

**DOI:** 10.3390/ma18020405

**Published:** 2025-01-16

**Authors:** Qiqi Hou, Zhongzhuang Zhang, Yaning Zhao, Kaiwei Ye, Jiajia Tian, Yuandong Mu, Jian He, Guotian Ye

**Affiliations:** 1 Henan Key Laboratory of High Temperature Functional Ceramics, School of Materials Science and Engineering, Zhengzhou University, Zhengzhou 450001, China; 17755199322@163.com (Q.H.); z13193572685@163.com (Z.Z.); zhao15733598803@163.com (Y.Z.); yehouhou2022@163.com (K.Y.); 980670097@163.com (J.T.); 2 China Construction Seventh Engineering Division Co., Ltd., Zhengzhou 450004, China; 3 Jiangsu Jingxin New Materials Co., Ltd., Yangzhou 225000, China; hj@jsjxgf.com

**Keywords:** alumina-rich spinel, exsolution, CAC-bonded corundum castables, interspersed interlocking structure, mechanical strength

## Abstract

This study investigates the effect of the exsolution behavior of alumina-rich spinel on the formation and distribution of CA_6_ (CaAl_12_O_19_) in corundum castables bonded with calcium aluminate cement. In this study, alumina-rich spinel is substituted for tabular corundum in the same proportions and grain size. The matrices after curing were analyzed by X-ray diffraction (XRD), scanning electron microscopy (SEM), and energy dispersive spectroscopy (EDS). The phase composition and microstructure of the matrices containing alumina-rich spinel were analyzed after firing at 1600 °C. These results showed that the addition of alumina-rich spinel significantly improved the mechanical strength of the castables. This improvement was attributed to the alumina produced by spinel exsolution during firing at 1600 °C, which reacted in situ with CA_2_ (CaAl_4_O_7_) to form CA_6_. CA_6_ connects the different particles and forms an interspersed interlocking structure within the spinel. The CA_6_-MA interspersed interlocking structure replaces part of the CA_6_-Al_2_O_3_ structure and significantly improves the mechanical strength of the castables.

## 1. Introduction

Corundum-based castable is a type of unshaped refractory material [1], which typically exhibits outstanding mechanical strength and high-temperature performance [2,3,4,5,6]. Consequently, it finds extensive applications in high-temperature furnaces for the iron and steel, non-ferrous metal, glass, cement, and other industries [7,8]. With the rapid development of the steel industry, corundum-based castables with higher thermal shock resistance are required for ladle linings. To improve the thermal shock resistance of castables, a certain amount of magnesia–alumina spinel is usually added to the castables [9,10].

Magnesia–alumina spinel exhibits a high melting point, a low coefficient of thermal expansion, high resistance to slag, and low thermal conductivity [11,12,13,14]. The typical formula of magnesia–alumina spinel is MgAl_2_O_4_. However, the magnesia–alumina spinel is actually a limited solid solution of MgO and Al_2_O_3_ [15]. In addition, the magnesia–alumina spinel has a wide solid solution range for alumina at 1600 °C, ranging from 71 wt.% to 82 wt.% according to the magnesia–alumina phase diagram [16]. When the Al_2_O_3_ content of spinel is higher than 82 wt.% at 1600 °C, the spinel could undergo exsolution.

Calcium aluminate cement (CAC) serves as the primary binder for corundum castables due to its excellent strength and mechanical strength at high temperatures [17,18,19,20,21]. The main mineral phases of high-alumina CAC are CA (CaAl_2_O_4_) and CA_2_ (CaAl_4_O_7_). When water comes in contact with the surface of CAC particles, these mineral phases begin to hydrate, resulting in various hydration products such as C_3_AH_6_ (Ca_3_Al_2_O_6_·6H_2_O), AH_3_ (Al_2_O_3_·3H_2_O), CAH_10_ (CaAl_2_O_4_·10H_2_O), and C_2_AH_8_ (Ca_2_Al_2_O_5_·8H_2_O) [22,23,24,25]. As the heating temperature rises, these hydration products gradually dehydrate and react with Al_2_O_3_ in castables to form CA_2_ at 1200 °C, followed by the generation of CA_6_ (CaAl_12_O_19_) at above 1400 °C. CA_6_ is usually present in hexagonal flakes, and it binds the corundum phase to provide strength to the castable [26,27,28]. However, when spinel is introduced into the CAC-bonded corundum castables, if the Al_2_O_3_ content of spinel is higher than 82 wt.%, the spinel will exsolve Al_2_O_3_ during calcination. Then, the exsolved Al_2_O_3_ may preferentially be involved in generating CA_6_, which in turn changes the distribution of CA_6_ in the castables, thus affecting the performance of the castables.

Numerous studies have demonstrated the beneficial effects of spinel addition on the properties of high-alumina refractories. For instance, Auvray et al. reported that incorporating spinel significantly enhances the corrosion resistance of high-alumina refractories [28]. Similarly, Diazl A observed that the addition of magnesia-rich spinel not only improved the strength of magnesia–spinel refractories but also increased their corrosion resistance, attributed to the in situ reaction between spinel and alumina leading to the formation of additional spinel [29]. This improvement is partially due to the volumetric expansion of spinel during its formation, which helps to fill the microscopic pores within the material, thereby enhancing its densification. Furthermore, Quan Z demonstrated that varying amounts of AR74 spinel (the spinel containing 74 wt.% alumina) could improve the corrosion resistance of Al_2_O_3_-SiO_2_-CaCO_3_ castables to different extents, further validating spinel’s effectiveness in enhancing the performance of refractory materials [30].

However, most of their studies focus on spinel with less than 82 wt.% alumina content, and the influence of spinel exsolution on the microstructure and mechanical properties of castable in the actual working environment is rarely investigated [6,31,32,33,34]. This work mainly aims to study the effect of the exsolved Al_2_O_3_ from MgAl_2_O_4_ on the formation and distribution of CA_6_ in CAC-bonded corundum–spinel castables. Various proportions (0, 5, 10, and 15 wt.%) of the spinel powders were added into CAC-bonded corundum-based castables, and the mechanical strength of castables and the phases and microstructure evolution of the matrix were investigated.

## 2. Materials and Methods

### 2.1. Raw Materials and the Preparation of Castables and Matrix

**Figure 1:**
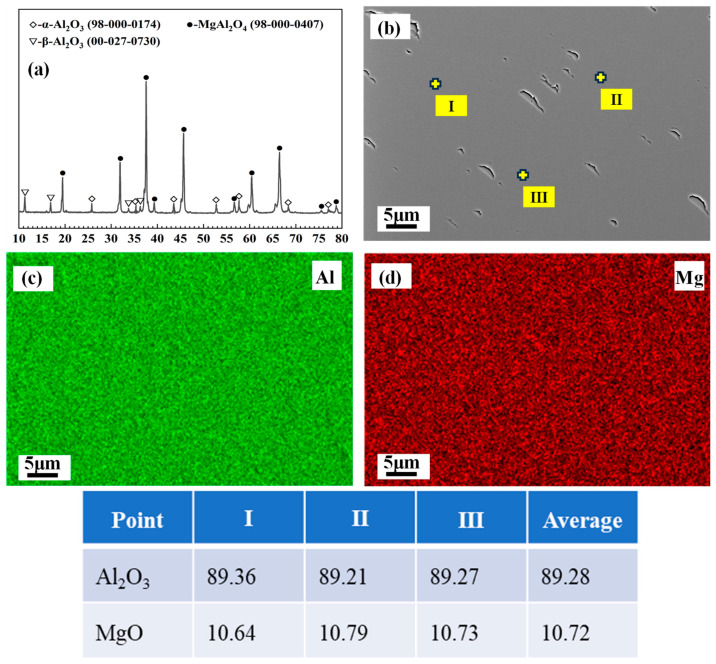
XRD (**a**) and EDS (**b–d**) analysis of MA-90.

The fused magnesia–alumina spinel powders were supplied by Henan Hecheng Special Refractory Materials Co., Ltd. (Kaifeng, China). The theoretical alumina content of this spinel is 90 wt.%, which is far beyond the alumina proportion (72 wt.%) of stoichiometric spinel. The XRD and EDS analysis in Figure 1 demonstrates that this spinel contains a small proportion of α- and β-alumina, and the actual alumina content in the spinel phase is about 89.28 wt.%. The presence of alumina phases is attributed to the impurities in the raw materials and the slight exsolution of alumina from the spinel during cooling. As the actual alumina content of this spinel is much closer to 90 wt.%, it is still marked as MA-90 in this paper.

**Figure 2:**
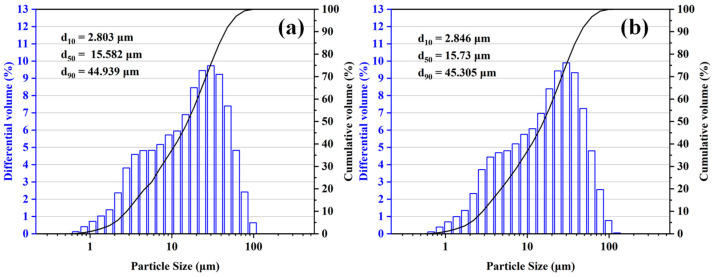
Particle size distribution of 325 mesh tabular alumina (**a**) and 325 mesh MA-90 (**b**).

In the present work, corundum–spinel castable was prepared using tabular alumina (6-3 mm, 3-1 mm, 1-0.5 mm, 0.5-0 mm, Jiangsu Jingxin New Materials Co., Ltd., Yangzhou, China) as aggregate. The tabular alumina fine powders (≤45 μm, Jiangsu Jingxin New Materials Co., Ltd.), fused MA-90 fine powders (≤45 μm), calcium aluminate cement (CAC, Secar 71, Kerneos Aluminate Technology Co., Ltd., Tianjin, China), and reactive alumina micro-powders (HA115, Jiangsu Jingxin New Materials Co., Ltd) form the matrix. The particle size distribution of fused MA-90 fine powders and tabular alumina fine powders used in this study is shown in Figure 2. The chemical composition of the tabular alumina used in this study is shown in Table 1. The total water addition was 4 wt.%. In addition, ADS1 and ADW1 (Qingdao Almatis Co., Ltd., Qingdao, China) were selected as dispersants.

The formulations of castables with different contents of spinel are shown in Table 2. The weighed raw materials were dry-mixed in a laboratory mixer for 1 min and then wet-mixed for another 2 min. Subsequently, the mixtures were cast into molds with sizes of 40 × 40 × 160 mm under vibration and then cured at 25 °C for 24 h. After curing, the castables were demolded for drying at 110 °C for 24 h and then fired at 1600 °C for 3 h.

The matrices were prepared according to the formulations in Table 3. The mixture was cast into a cylindrical specimen with Φ 50 × 50 mm. After curing at 25 °C for 24 h, the specimens were demolded. And they were dried at 110 °C for 24 h and fired at 1600 °C for 3 h.

### 2.2. Methods

The cold modulus of rupture (CMOR) and cold compressive strength (CCS) of castables after drying and firing were determined in accordance with the Chinese standards GB/T 3001-2007 [35] and GB/T 5072-2023 [36], respectively. CMOR is measured using a three-point bending test, with the formula CMOR = $\frac{3FL}{2bd^{2}}$. Among them, F is the maximum load, L is the distance between the support points, b is the width of castables, and d is the thickness of castables. CCS is determined through a compression test, with the formula CCS = $\frac{F}{A}$. Among them, F is the maximum load, and A is the stress area of castables.

The castables after firing at 1600 °C were used in the thermal shock resistance test according to the Chinese standard YB/T 4018-1991 [37]. The castables were held at 1100 °C for 30 min and then cooled to 25 °C in air for one cycle. After repeating the above cycle 3 times, the CMOR of the castables was tested. The residual CMOR was used to characterize the thermal shock resistance of castables with various contents of spinel. The mean of three samples was taken to determine the CCS, CMOR, and the remaining CMOR values.

XRD was used to analyze the phase compositions of MA-90 before and after calcination at 1600 °C for 3 h. Furthermore, the matrix samples’ composition was analyzed following firing at 1600 °C for 3 h. XRD analysis was conducted using a Bruker D8 Advance diffractometer (Bruker, Ettlingen, Germany) at a scanning speed of 2° min^−1^ in the 2θ range from 10° to 80°. Semiquantitative XRD (SXRD) phase analysis of the matrices after firing was performed using the Rietveld method with Topas 6.0 software (Bruker, Germany) by refining parameters and fitting the peak areas.

The matrices after firing were embedded in resin and then polished using a metallographic polishing machine after solidification. The cross-section microstructure of the matrices with different contents of spinel was characterized by means of a scanning electron microscope (ZEISS, Oberkochen, Germany), equipped with energy-dispersive X-ray spectroscopy (Oxford Instruments, Abingdon, UK).

## 3. Results and Discussion

### 3.1. Exsolution of MA-90

**Figure 3:**
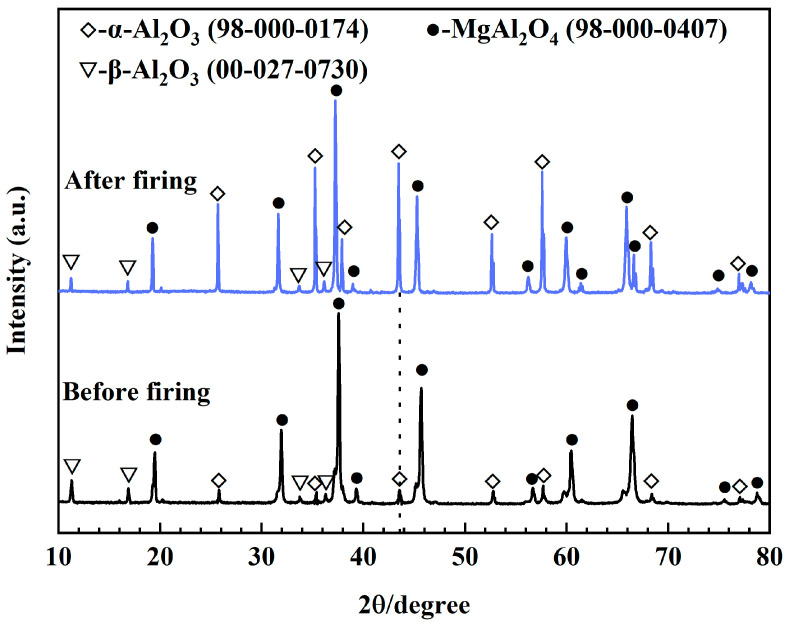
XRD patterns of MA-90 before and after firing at 1600 °C for 3 h.

Figure 3 shows the XRD patterns of MA-90 before and after firing at 1600 °C for 3 h. The crystal phases in MA-90 contain MgAl_2_O_4_ (MA), α-Al_2_O_3_, and β-Al_2_O_3_ before firing. After firing at 1600 °C, the predominant phases remain α-Al_2_O_3_, MA, and β-Al_2_O_3_. Furthermore, the diffraction peaks of β-Al_2_O_3_ in the sample have shown no significant change, while the diffraction peaks of α-Al_2_O_3_ at 25.67°, 35.37°, 43.54°, 52.76°, 57.69°, and 68.30° are significantly higher. R_wp_ (weighted profile R factor) is a parameter used in semi-quantitative XRD analysis to evaluate the quality of the fit between observed and calculated diffraction patterns, with lower values indicating a better fit. The semiquantitative analysis in Table 4 demonstrates that the content of α-Al_2_O_3_ in the sample increases from 6.3 wt.% to 42.9 wt.% after firing, demonstrating that MA-90 undergoes considerable exsolution after firing at 1600 °C.

### 3.2. Mechanical Strength

**Figure 4:**
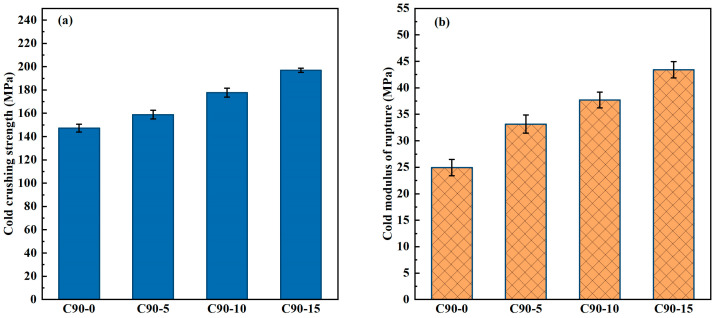
Cold crushing strength (**a**) and cold modulus of rupture (**b**) of corundum–spinel castable after firing at 1600 °C for 3 h.

It can be postulated that exsolution of MA-90 after firing may impact the performance of the castables. In order to ascertain the effect of MA-90 exsolution on the mechanical strength of the castables, an investigation was conducted to determine the CMOR and CCS of the castables with varying contents of MA-90. As spinel powders were introduced by replacing the same proportion and particle size of tabular corundum powders, the flowability of the four groups of castables was basically similar. Therefore, the flowability of the castables has a fundamentally similar effect on the mechanical strength of the castables. Figure 4 presents the CMOR and CCS of castables added with MA-90 after firing at 1600 °C. As the MA-90 content is raised from 0 to 15 wt.%, the CMOR and CCS of castables after firing at 1600 °C increase. For instance, the CCS of castables improves from 147.3 MPa to 196.9 MPa as the MA-90 content increases from 0 to 15 wt.%. Meanwhile, the CMOR of castables grows by 74.3% from 24.9 MPa to 43.4 MPa. In summary, the addition of MA-90 can markedly improve the mechanical strength of CAC-bonded corundum castables.

### 3.3. Thermal Shock Resistance

**Figure 5:**
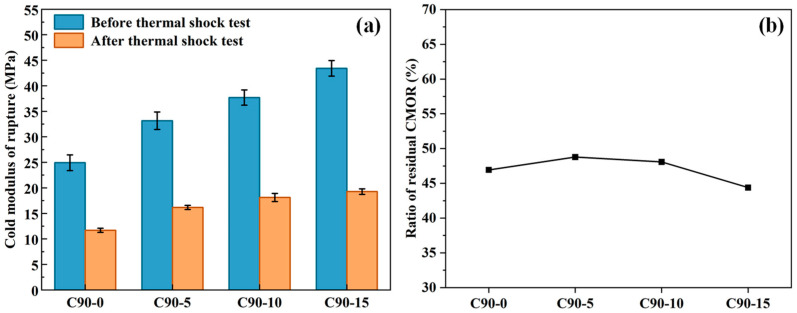
Effect of MA-90 content on thermal shock resistance of corundum–spinel castable after heat-treating at 1100 °C: (**a**) CMOR before and after thermal shock test; (**b**) ratio of residual CMOR.

The CMOR and ratio of residual CMOR of pre-treated specimens after firing are shown in Figure 5a,b. As shown in Figure 5a, the residual flexural strength of the castables increased slightly with the addition of MA-90. For example, the residual flexural strength of the control sample is 11.70 MPa, while the residual flexural strength of the castables containing 5 wt.% MA-90 is 16.17 MPa, resulting in a 38.2% increase. With the spinel content increased to 15 wt.%, the residual flexural strength of the castables improved to 19.27 MPa, which represents a 64.7% increase. However, as shown in Figure 5b, the residual flexural strength retention of the castables always remained at about 47%.

This may be due to the difference in thermal expansion coefficients between spinel (8.9 × 10^−6^ °C^−1^) and corundum (8.3 × 10^−6^ °C^−1^) at high temperatures; microcracks formed between the two interfaces during the rapid cooling of thermal shock resistance tests, resulting in reduced flexural strength. With the spinel content increased, more and more microcracks could be generated in the castables after the thermal shock test, so the castables’ flexural strength decreased significantly. It can be seen from Figure 5b that the ratio of residual CMOR slightly decreases as the spinel content increases from 0 to 15 wt.%. This also indicates that the addition of an excessive amount of MA-90 is not beneficial to the thermal shock resistance of the castables.

### 3.4. Phase Composition

**Figure 6:**
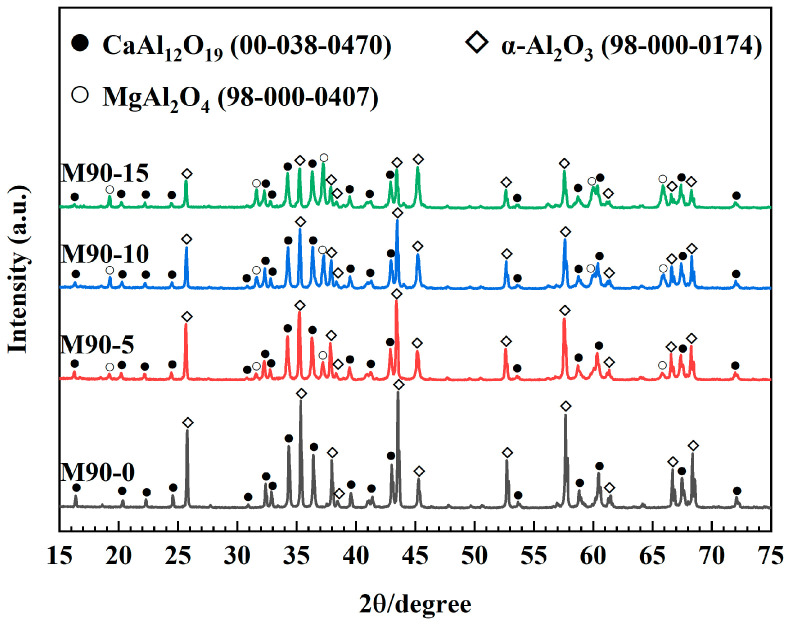
XRD patterns of the matrices after firing at 1600 °C.

**Figure 7:**
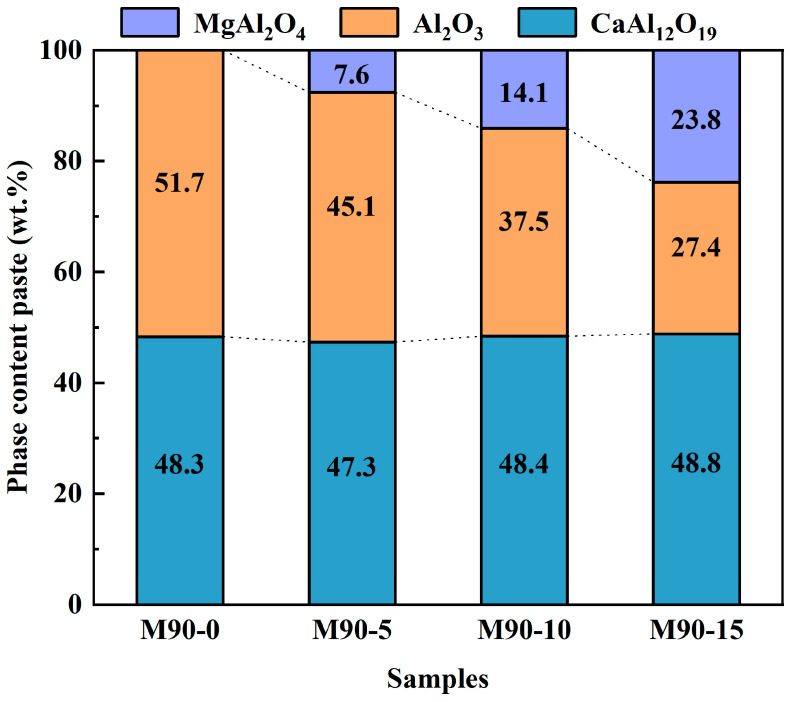
Phase composition of the matrices after firing at 1600 °C.

Figure 6 and Figure 7 illustrate the XRD patterns and the results of semiquantitative analyses of matrices with varying MA-90 contents after firing at 1600 °C. As illustrated in Figure 6, the predominant phases in the control sample are corundum (Al_2_O_3_) and CaAl_12_O_19_ (CA_6_). And the main phases in M90-5, M90-10, and M90-15 are Al_2_O_3_, CA_6_, and MgAl_2_O_4_ (MA).

To determine the percentage of phase composition in each type of matrix, the XRD patterns shown in Figure 6 were analyzed using Rietveld refinement. The R_wp_ values of M90-0, M90-5, M90-10, and M90-15 are 14.22%, 13.6%, 14.53%, and 14.18%, respectively. Figure 7 shows that all specimens have a uniform CA_6_ content of about 47–49%. Since calcium aluminate cement is the only source of calcium, it entirely converts to CA_6_ upon firing at 1600 °C. According to Table 4, the amount of calcium aluminate cement in each sample is the same, and the amount of CA_6_ produced is equal. According to Figure 7 and Table 4, it can be calculated that the content of Al_2_O_3_ in the matrices decreases after firing. For example, the formation of CA_6_ in the control sample consumed 35.5% of Al_2_O_3_, while M90-5, M90-10, and M90-15 consumed 24.9%, 16%, and 9.6% of Al_2_O_3_, respectively. With the increase in MA content, the amount of corundum consumed in the matrix gradually decreases. This also indicates that the MA exsolves Al_2_O_3_ from the interior and replenishes the corundum in the matrix after firing. A portion of Al_2_O_3_ required to generate CA_6_ is derived from the matrix composition with spinel, while the exsolution of MA-90 provides the remainder. This result also proves that spinel exsolution occurred after firing at 1600 °C.

According to Figure 7 and Table 4, it can also be calculated that the spinel content in M90-5, M90-10, and M90-15 is reduced by 9.4%, 19.4%, and 22.6%, respectively. As the spinel content increases, more Al_2_O_3_ is exsolved from spinel.

In conclusion, the Al_2_O_3_ exsolved from MA participated in generating CA_6_. With the increase in MA addition, the content of exsolved Al_2_O_3_ in MA increased. Furthermore, the exsolution of Al_2_O_3_ from MA to generate CA_6_ is also increasing gradually.

### 3.5. Microstructure of the Matrix

**Figure 8:**
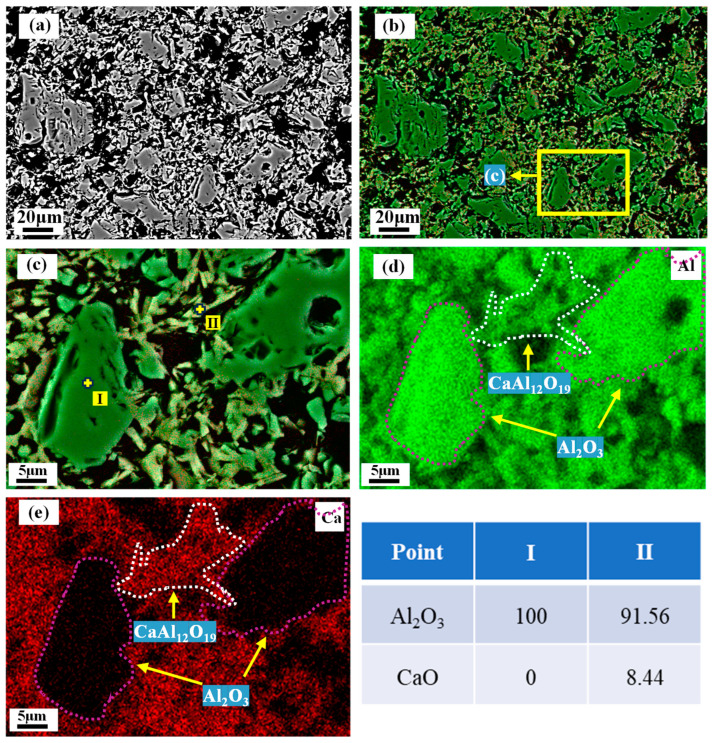
Phase distribution of M90-0 after firing at 1600 °C. (**a**) BSE image, (**b**,**c**) composite SE-EDS image, (**d**,**e**) EDS image.

It has been established that MA exsolves Al_2_O_3_ after firing. Then, the exsolved Al_2_O_3_ may react with CA_2_ to form CA_6_, which is most likely to change the internal microstructure of the castables. Therefore, the microscopic morphology of the matrix with different MA-90 contents added is observed, which in turn elucidates the reasons affecting the properties of the castables. Figure 8a illustrates the microscopic morphology of M90-0 after firing at 1600 °C. Figure 8c shows that Point (I) is corundum and Point (II) is CA_6_ as analyzed by EDS. After firing at 1600 °C, CA_6_ and corundum sintered together to form a CA_6_-Al_2_O_3_ structure. Figure 8c shows that CA_6_ primarily occurs between the corundum particles, forming a structure providing strength to the castable.

**Figure 9:**
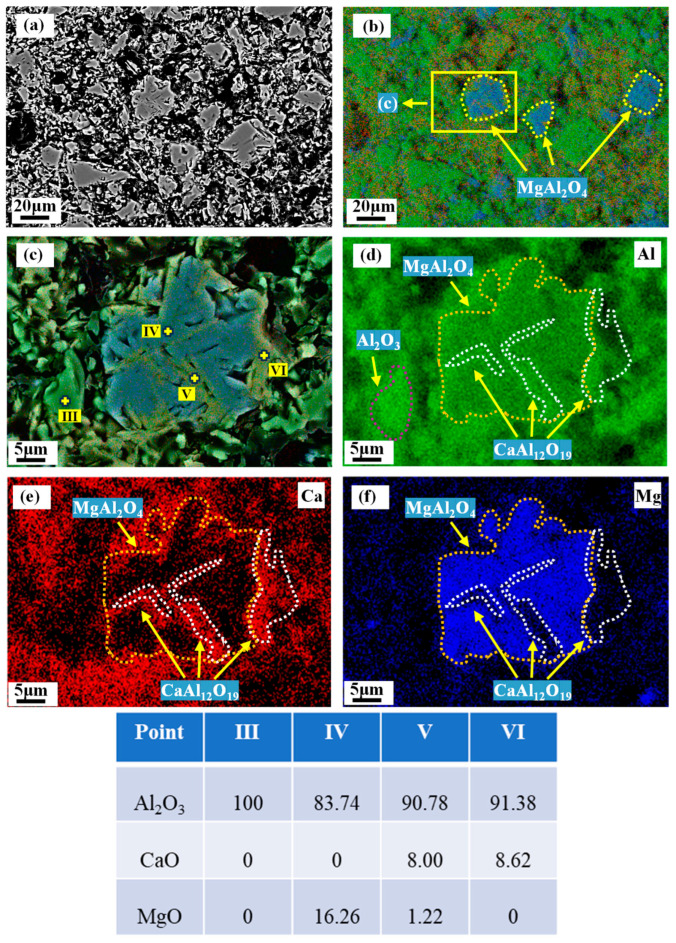
Phase distribution of M90-5 after firing at 1600 °C. (**a**) BSE image, (**b**,**c**) composite SE-EDS image, (**d**–**f**) EDS image.

Figure 9a shows the phase distribution of M90-5 after firing at 1600 °C. The results of EDS analysis show that Point (III) is Al_2_O_3_, Point (IV) is MgAl_2_O_4_, and Points (V) and (VI) are CA_6_. As illustrated in Figure 9c, a portion of CA_6_ is generated at the edge of the spinel, and another portion of CA_6_ traverses the interior of the MA, forming a CA_6_-MA interspersed interlocking structure. This can be attributed to the exsolution of Al_2_O_3_ from MA-90 during firing at 1600 °C. The exsolved alumina has higher reactivity, which reacts preferentially with calcium oxide, to form in situ the interspersed CA_6_ within the MA.

As demonstrated in Figure 9c, the Al_2_O_3_ content of MA-90 decreased from 89.28 wt.% to 82.9 wt.%, indicating that the MA undergoes exsolution after firing at 1600 °C. Furthermore, there is no direct link between corundum and spinel in Figure 9c. It is worth noting that the CA_6_-MA interlocked structure is tighter than the CA_6_-Al_2_O_3_ structure. The CA_6_-MA interspersed interlocking structure replaced part of the CA_6_-Al_2_O_3_ structure when 5 wt.% spinel was added to the castables. Consequently, the cold strength of C90-5 is higher than that of C90-0.

**Figure 10:**
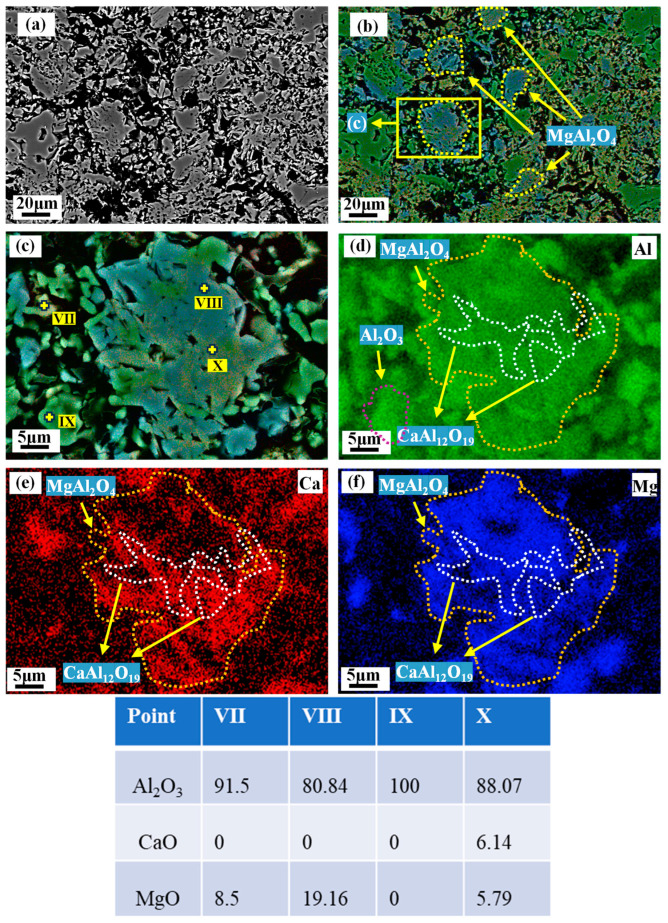
Phase distribution of M90-10 after firing at 1600 °C. (**a**) BSE image, (**b**,**c**) composite SE-EDS image, (**d**–**f**) EDS image.

Figure 10a depicts the microscopic morphology of M90-10 after firing at 1600 °C. Based on the EDS analysis, it can be deduced that Point (VII) is CA_6_, Point (IX) is Al_2_O_3_, and Point (VIII) is MgAl_2_O_4_. Point (X) demonstrates that CA_6_ is interspersed within MA. In addition, the alumina content of MA-90 decreased from 89.28 wt.% to 82.4 wt.%. This suggests that the alumina exsolution occurs within the alumina-rich spinel after firing. As illustrated in Figure 10b, the CA_6_-MA interspersed interlocking structure in the matrix gradually increases with the increase in MA content.

**Figure 11:**
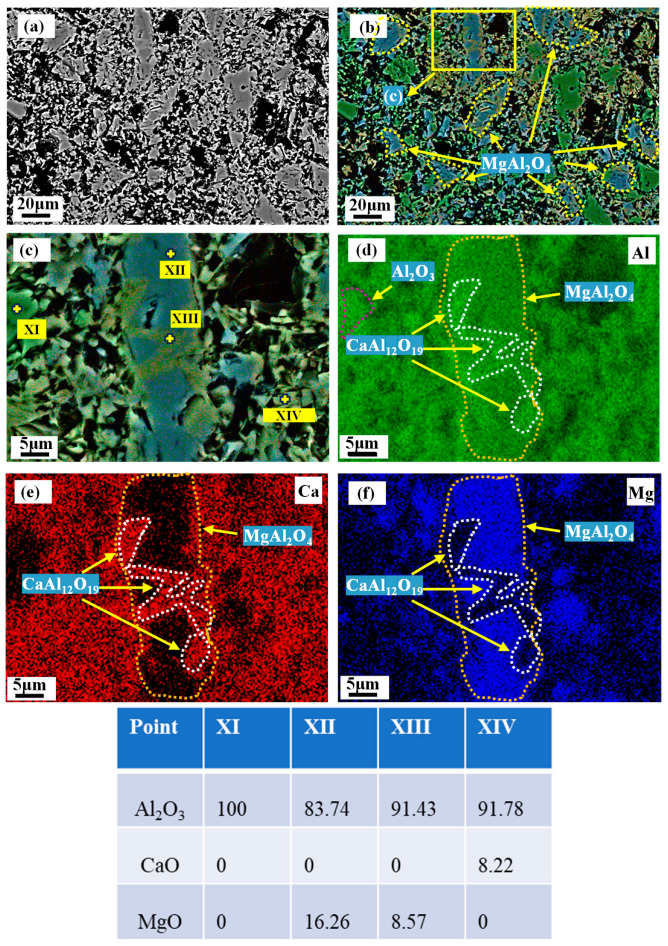
Phase distribution of 90-15 matrix after firing at 1600 °C. (**a**) BSE image, (**b**,**c**) composite SE-EDS image, (**d**–**f**) EDS image.

Figure 11a depicts the microscopic morphology of M90-15 after firing at 1600 °C. Based on the EDS analysis, the principal phases present in the M90-15 matrix specimen are Al_2_O_3_, MA, and CA_6_. Figure 11c shows that CA_6_ is generated in MA, forming a CA_6_-MA interspersed interlocking structure.

As MA content in the castables increases, more alumina has been exsolved from MA. More CA_6_-MA interspersed interlocking structures are generated in the matrix. Comparison of Figure 9b and Figure 10b with Figure 11b shows an increase in the replacement of the CA_6_-Al_2_O_3_ structure by the CA_6_-MA interspersed interlocking structure in Figure 11b, which may have made the cold strength of C90-15 much higher than that of C90-5 and C90-10.

### 3.6. Mechanism of CA_6_ Formation and Distribution

**Figure 12:**
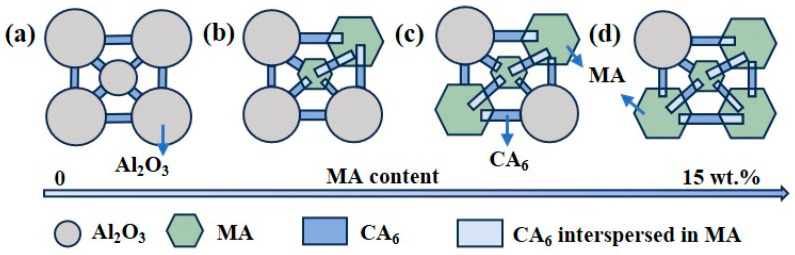
Schematic of structure formation of CA_6_ in a matrix containing different proportions of alumina-rich spinel. (**a**) M90-0 (**b**) M90-5 (**c**) M90-10 (**d**) M90-15.

Based on the above analysis, the mechanism of CA_6_ formation and distribution of CAC-bonded corundum castables containing alumina-rich spinel can be represented as Figure 12. Figure 12a shows the schematic microstructure of the sample matrix without MA addition, mainly consisting of CA_6_-Al_2_O_3_ structures. Figure 12b illustrates the castable’s microstructure after introducing a small quantity of MA-90. One portion of CA_6_ is attached to corundum particles, while the other portion is irregularly dispersed within MA, forming a CA_6_-MA interspersed interlocking structure.

This phenomenon is attributed to the fact that the exsolution temperature of alumina-rich spinel is close to the formation temperature of CA_6_. When the firing temperature rises to 1400 °C, spinel begins to exsolve alumina. Simultaneously, CA_2_ reacts with the alumina to form CA6. Due to the higher reactivity of the exsolved alumina, it preferentially reacts with CA_2_, resulting in the uniform dispersion of the generated CA_6_ within the spinel particles. The CA_6_-MA interspersed interlocking structure provides critical support and load-bearing functions during strength testing, effectively distributing external loads and enhancing the castables’ overall strength. Consequently, as the MA content gradually increases, the CA_6_-MA interspersed interlocking structure also increases accordingly. The CA_6_-MA interspersed interlocking structure gives the castable greater mechanical strength. With a further increase in spinel content in the castables, as shown in Figure 12c,d, the CA_6_-MA interspersed interlocking structure gradually replaces the CA_6_-Al_2_O_3_ bonded structure, which significantly improves the post-firing strength of the castables.

## 4. Conclusions

The alumina-rich spinel exsolves Al_2_O_3_ after calcination at 1600 °C, resulting in a decrease in Al_2_O_3_ content from 89.28 wt.% to 82 wt.%. With the addition of MA-90 spinel to the castables, the exsolved alumina has higher reactivity, which reacts preferentially with CA_2_ to form CA_6_. As a result, CA_6_ is irregularly interspersed within the spinel particles, forming a CA_6_-MA interspersed interlocking structure.

The CA_6_-MA interspersed interlocking structure is tighter than the CA_6_-Al_2_O_3_ structures. When the CA_6_-MA interspersed interlocking structure replaces part of the CA_6_-Al_2_O_3_ structure, it enhances the mechanical strength of the castables. As the quantity of alumina-rich spinel incorporated into the castables increases, the content of exsolved alumina rises, forming more and more CA_6_-MA interspersed interlocking structures and improving the mechanical strength of the castables.

## Figures and Tables

**Table 1:** Chemical composition of tabular alumina used in this work (wt.%).

Al_2_O_3_	SiO_2_	Fe_2_O_3_	Na_2_O
99.56	0.09	0.05	0.30

**Table 2:** Formulations of castables with different contents of spinel (wt.%).

Raw Materials		Castable Samples			
		C90-0	C90-5	C90-10	C90-15
Tabular alumina	6-3 mm	28	28	28	28
	3-1 mm	22	22	22	22
	1-0 mm	20	20	20	20
	d_50_ = 15.582 μm	20	15	10	5
Reactive alumina	HA115	6	6	6	6
Cement	Secar 71	4	4	4	4
MA-90	d_50_ = 15.73 μm	0	5	10	15
Dispersant	ADW1	+0.4	+0.4	+0.4	+0.4
	ADS1	+0.1	+0.1	+0.1	+0.1

**Table 3:** Formulations of matrices with different contents of spinel (wt.%).

Raw Materials		Matrix Samples			
		M90-0	M90-5	M90-10	M90-15
Tabular alumina	d_50_ = 15.582 μm	67	50	33.5	17
Reactive alumina	HA115	20	20	20	20
Cement	Secar 71	13	13	13	13
MA-90	d_50_ = 15.73 μm	0	17	33.5	50
Dispersant	ADW1	+0.4	+0.4	+0.4	+0.4
	ADS1	+0.1	+0.1	+0.1	+0.1

**Table 4:** The percentage of phase composition in MA-90 obtained from semiquantitative analysis.

Sample	Phase Quantification/wt.%			Agreement Factors
	MA	α-Al_2_O_3_	β-Al_2_O_3_	R_WP_/%
Before firing	88.3	6.3	5.4	11.47
After firing	51.3	42.9	5.8	12.02

## Data Availability

The original contributions presented in this study are included in the article. Further inquiries can be directed to the corresponding author.
